# Amyloid Fibrils Produced by *Streptococcus sanguinis* Contribute to Biofilm Formation and Immune Evasion

**DOI:** 10.3390/ijms242115686

**Published:** 2023-10-28

**Authors:** Eduardo M. Franco, Lívia A. Alves, Hassan Naveed, Victor A. A. Freitas, Débora C. Bastos, Renata O. Mattos-Graner

**Affiliations:** 1 Department of Oral Diagnosis, Piracicaba Dental School, State University of Campinas, Piracicaba 13414-903, SP, Brazil; eduardomfranco@live.com (E.M.F.); liviaaalves@hotmail.com (L.A.A.); hassankhan5293@gmail.com (H.N.); victoraragao.mn@gmail.com (V.A.A.F.); 2 School of Dentistry, Cruzeiro do Sul University (UNICSUL), São Paulo 01506-000, SP, Brazil; 3 Department of Biosciences, Piracicaba Dental School, State University of Campinas, Piracicaba 13414-903, SP, Brazil; bastosdc@unicamp.br; 4 São Leopoldo Mandic Medical School, Campinas 13045-755, SP, Brazil

**Keywords:** *Streptococcus sanguinis*, amyloid fibrils, biofilm, complement system, neutrophil, fitness, human blood, EGCG, systemic virulence

## Abstract

Bacterial surface proteins assembled into amyloids contribute to biofilm formation and host immune evasion. *Streptococcus sanguinis*, a pioneer colonizer of teeth commonly involved in cardiovascular infections, expresses about thirty-three proteins anchored to the cell wall by sortase A. Here, we characterized the production of amyloid in *S. sanguinis* strains differing in biofilm and immune evasion phenotypes and investigated the role of sortase A in amyloidogenesis. Amyloid was identified in biofilms formed by nine strains, using Congo red (CR) staining and cross-polarized light microscopy. Additionally, EGCG, an amyloid inhibitor, impaired biofilm maturation in a strain-specific fashion. The amounts of amyloid-like components quantified in culture fluids of nine strains using thioflavin T and fluorimetry negatively correlated with bacterial binding to complement-activating proteins (SAP, C1q), C3b deposition and rates of opsonophagocytosis in PMNs, implying amyloid production in immune evasion. The deletion of the sortase A gene (*srtA*) in strain SK36 compromised amyloid production and sucrose-independent biofilm maturation. The *srtA* mutant further showed increased susceptibility to C3b deposition and altered interactions with PMNs as well as reduced persistence in human blood. These findings highlight the contribution of amyloids to biofilm formation and host immune evasion in *S. sanguinis* strains, further indicating the participation of sortase A substrates in amyloidogenesis.

## 1. Introduction

*Streptococcus sanguinis* is a pioneer commensal colonizer of tooth surfaces of humans, which promotes the homeostasis of dental biofilms by inhibiting the colonization by oral pathogens, such as *Streptococcus mutans* and *Porphyromonas gingivalis* [1,2]. On the other hand, *S. sanguinis* is commonly involved in infective endocarditis (IE) in susceptible hosts, and eventually, in immunocompetent young adults [3,4,5,6]. This species is also frequently detected in atheromatous plaques, further indicating a particular capacity to promote cardiovascular infections [6]. As compared to other streptococci of the oral cavity and oropharynx, the *S. sanguinis* genome harbors a remarkable number of cell surface proteins [7]. These include thirty-three proteins with an LPxTG motif recognized by sortase transpeptidases, which anchors target precursor proteins to the peptideoglycan [7,8]. When compared to other commensal streptococci, *S. sanguinis* further shows increased capacity to evade immune components abundant in whole saliva and in gingival crevicular fluid, which include proteins of the complement system, a major defense arm of the cardiovascular system [9,10]. The large number of cell surface proteins expressed by *S. sanguinis* might underlie its increased competitiveness for colonizing the dental pellicle (formed mostly by salivary and host tissue components) [11] as well as extra-oral tissues. However, the molecular mechanisms involved in *S. sanguinis* contribution to initial and maturing phases of biofilm formation in dental surfaces and/or cardiovascular tissues must still be better understood.

*Streptococcus sanguinis* ability to produce H_2_O_2_ under O_2_ availability contributes to the initial phases of biofilm formation, because H_2_O_2_ inhibits competitor species, e.g., the caries pathogen *Streptococcus mutans* [12,13], further promoting the lytic-independent production of extracellular DNA (eDNA) [14], which is required for biofilm initiation [15]. Under sucrose availability, *S. sanguinis* also synthesizes glucan, but these exopolysaccharides do not confer sufficient strength to biofilms at maturing phases, due to their reduced stability when compared to glucan produced by *S. mutans* [12,15,16]. Binding to sucrose-derived glucan is, on the other hand, significantly associated with *S. sanguinis* evasion to complement deposition in a strain-specific fashion [17], linking glucan-binding to *S. sanguinis* capacity to evade host immune functions. However, whether *S. sanguinis* strains produce additional extracellular components involved in glucan interaction, biofilm formation and/or host immune evasion remains to be addressed. 

Multiple eukaryotic and microbial proteins may be self-assembled into amyloid fibrils under certain conditions. Although in humans, different types of amyloid fibrils are associated with diseases [18], amyloid formation is currently recognized as a common physiological process in eukaryotic and prokaryotic organisms; thus, the term “functional amyloid” was assigned [19]. Generally, these protein-derived exopolymers are formed of layers of repetitive cross β-sheet organized perpendicularly to the axis of the maturing fibril, a conformation responsible for unique traits of amyloid fibrils regardless of the type of the protein sub-unit. These include proteolytic resistance and affinity to thioflavin T (ThT) and to Congo red (CR) dyes [20,21,22]. Importantly, functional amyloids produced by microorganisms have functions in the adhesion to and invasion of host tissues, in biofilm maturation, in binding to exopolysaccharides, and in the evasion of the host immune system [20,21,22]. In this study, we investigated the production of functional amyloid by *S. sanguinis* strains and explored its roles in biofilm formation and in evasion to complement-mediated immunity. In addition, by analyzing an isogenic mutant of the sortase A gene (*srtA*), we addressed the potential roles of cell wall anchored proteins in amyloid production and related phenotypes of biofilm formation and virulence.

## 2. Results

### 2.1. Streptococcus sanguinis Strains Produce Amyloid Fibrils in Biofilms

**Figure 1:**
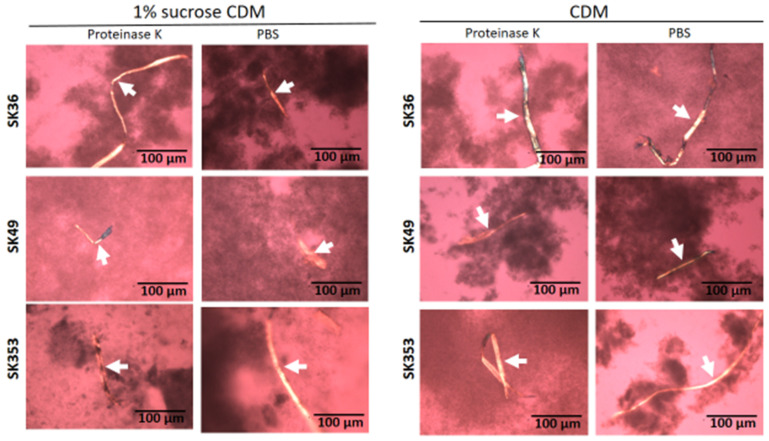
Detection of amyloid fibrils in *S. sanguinis* biofilms. Biofilms were formed by strains SK36, SK49 and SK353 in CDM with or without 1% sucrose over 72 h. After treatment with proteinase K or PBS (control), the biofilms were stained with CR and analyzed under cross-polarized light microscopy. Digital images of apple-green amyloid fibrils (arrows) were obtained using bright field microscopy and under a cross-polarized filter with the help of a coupled digital camera, Leica DMLP (200× total magnification). Strain identities are indicated to the left of the horizontal panels.

Amyloid fibrils are produced by *S. mutans* strains and seems to contribute to biofilm structure [23,24,25]. Thus, we assessed the presence of amyloid fibrils in biofilms formed on 24-well plates in chemically defined medium (CDM) supplemented or not supplemented with 1% sucrose in three *S. sanguinis* strains (SK36, SK49 and SK353), which were previously shown to have different biofilm phenotypes [17]. Because functional amyloids are typically resistant to proteases [26], biofilms analyzed were previously treated or not treated with proteinase K. For reference, the production of amyloid fibrils was also assessed in the *S. mutans* strains UA159 and OMZ175, which were previously shown to form amyloids in similar assays [25,27]. As shown in Figure 1, 72 h biofilms formed by all the three *S. sanguinis* strains showed typical proteinase K-resistant fibrous aggregates of approximately 10 µm in width, as detected by CR-staining and polarized light microscopy. In addition, although the biofilms formed in 1% sucrose CDM showed increased biomass when compared to biofilms formed in CDM, we were notable to detect differences either in the presence or amounts of amyloid fibrils between biofilms formed in the presence or absence of sucrose. As expected, fibril birefringence was not detected when samples were analyzed under bright field microscopy. Similar amyloid fibrils were also detected in biofilms formed by the *S. mutans* control strains UA159 and OMZ157. Therefore, mature biofilms of *S. sanguinis* strains produce protease-resistant amyloid-like fibrils, regardless of the presence of glucan synthesized from sucrose.

### 2.2. Streptococcus sanguinis Strains Produce Amyloid-like Components in Culture Fluids and in Macrocolonies

**Figure 2:**
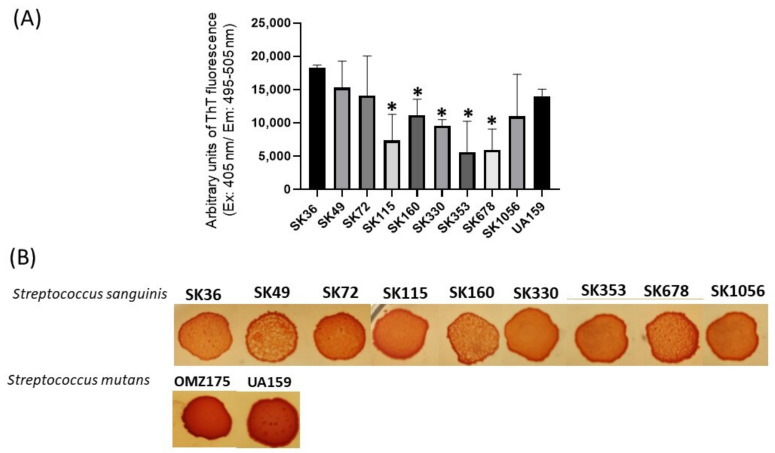
Profiles of amyloid-like components produced by *S. sanguinis* strains. (**A**) Samples of concentrated culture supernatants were incubated (4 °C; 48 h) for amyloid formation, and ThT was added to measure fluorescent intensities in a GloMax^®®^ Discover System (AFC filter; excitation: 405 nm; emission: 495–505 nm). Data were expressed as arbitrary units of ThT fluorescence. Columns indicate the mean of data obtained in three independent cultures; bars indicate standard deviation. Asterisks indicate significant difference in relation to the SK36 strain (Mann–Whitney U-test; *p* < 0.01). (**B**) CR-binding phenotypes of macrocolonies grown within 72 h on BHI agar supplemented with CR. Digital images of the macrocolonies were obtained under bright light. The *S. mutans* strains UA159 and OMZ175 were used as reference.

We next applied a fluorimetric assay to quantify amyloid-like components in samples of concentrated supernatants (1 µg/mL of protein) prepared from CDM cultures of nine *S. sanguinis* strains. As shown in Figure 2A, the culture supernatant samples of all the strains analyzed showed amyloid-like components emitting fluorescence in the presence of ThT. There was, however, significant diversity in the amounts of these components among strains, and particular strains (SK353 and SK678) produced reduced levels of amyloid-like components (Figure 2A). Because there is no streptococcal strain known to be devoid of amyloid production, which could be used under the same culture conditions, we used BSA (1 µg/mL) as negative controls in these quantitative assays. Amyloid production was further investigated in macrocolonies formed in BHI agar supplemented with CR (CR-BHI). As shown in Figure 2B, macrocolonies formed on CR-BHI agar by all the *S. sanguinis* strains sequestered CR from media, as observed via the formation of dark red edges. The two *S. mutans* reference strains (UA159 and OMZ175) formed darker macrocolonies when compared to *S. sanguinis* strains (Figure 2B). Thus, *S. sanguinis* produces amyloid-like components that bind to ThT and to CR, but there is diversity among strains regarding the amounts of these components in culture fluids. 

### 2.3. EGCG Inhibits Biofilm Formation by S. sanguinis at Specific Concentrations

**Figure 3:**
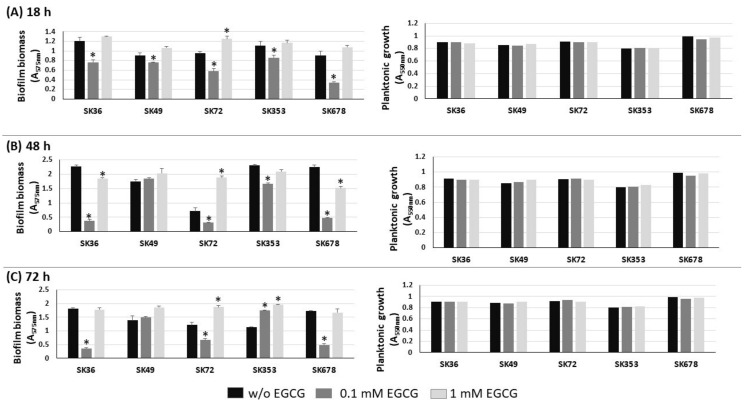
Effects of EGCG on biofilm formation by *S. sanguinis* strains. Biofilms were formed on 96-well plates in BHI 1% sucrose supplemented or not with EGCG for 18 (**A**), 48 (**B**) and 72 h (**C**) (at 37 °C; 10% CO_2_). The absorbances of the ethanol eluates of biofilms previously stained with crystal violet were expressed as indirect measures of biofilm biomass. Planktonic growth of the same cultures monitored at the same incubation periods (respective right panels in **A**–**C**). Columns represent means of triplicates of one representative experiment; bars indicate standard deviations. Asterisk indicates statistically significant differences in relation to the same strain grown in medium without EGCG (Kruskal–Wallis with Dunn’s post hoc test; *p* < 0.05).

Biofilm formation by *S. mutans* strains is significantly inhibited by EGCG, an inhibitor of amyloid production [23,28]. Therefore, we investigated the influence of EGCG on biofilm phenotypes of nine *S. sanguinis* strains after 18, 48 and 72 h of incubation in 1% sucrose BHI supplemented or not with EGCG. As shown in Figure 3, medium supplementation with 0.1 mM EGCG significantly inhibited biofilm formation in most strains at all the stages of biofilm maturation (18 to 72 h of growth), except for strain SK49. On the other hand, at a 10-fold higher concentration (1 mM), EGCG did not affect biofilm formation. Importantly, medium supplementation with EGCG at either 0.1 or 1 mM did not affect the planktonic growth of the strains (Figure 3, right panels). Strains did not grow in CDM supplemented with EGCG, precluding the analysis of EGCG on biofilm formation in this culture medium. Thus, EGCG significantly inhibits biofilm maturation in most *S. sanguinis* strains when at a specific concentration range in a complex BHI medium. 

### 2.4. Amyloid Production Negatively Correlates with Binding to Serum Amyloid P Component (SAP), C3b Deposition and Opsonophagocytosis by PMNs among S. sanguinis Strains

**Figure 4:**
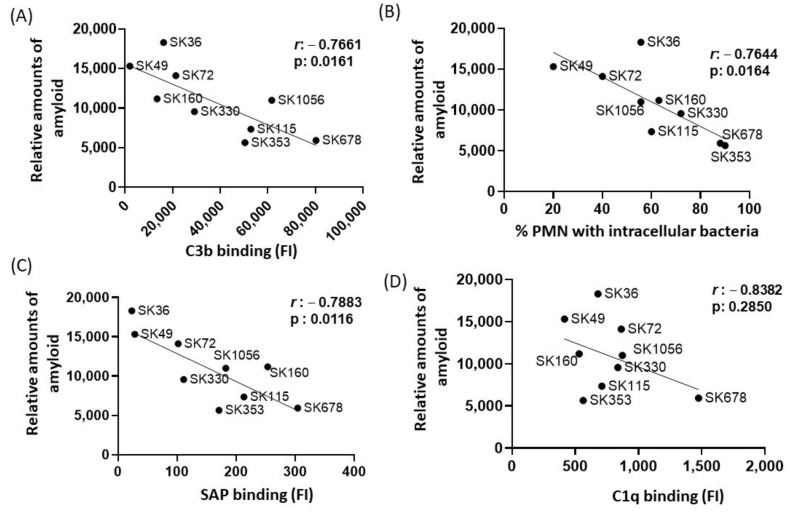
Correlation analysis between amyloid production and profiles of binding to complement proteins or opsonophagocytosis by PMNs. Pearson’s correlation was applied to analyze the associations between the intensities of binding to C3b (**A**), SAP (**C**) and C1q (**D**) or frequencies of opsonophagocytosis by PMNs (**B**) (determined in a previous study [17] with the amounts of amyloid detected in culture supernatants in nine *S. sanguinis* strains. The *r* coeficients and *p* values are shown for each correlation scatter plot.

In *E. coli*, functional amyloid curli increases bacterial survival in human serum, apparently through amyloid binding to C1q and the inhibition of the classical pathway of complement system [29]. Previously, we reported marked differences in binding to C3b and serum-mediated phagocytosis by PMNs among the same studied *S. sanguinis* strains, in a fashion associated with the strain-specific capacities of binding to major recognition proteins of the classical pathway, C1q and SAP [17]. Diversity in binding to the complement the downregulators C4-binding protein (C4BP) and factor H (FH) was also reported in the same strains [17]. Therefore, we compared the relative amounts of amyloid-like components quantified in culture fluids in the nine strains with previously published data of intensities of strain binding to C3b, C1q, SAP, C4BP and FH or frequency of opsonophagocytosis by PMNs [17]. By applying Pearson’s correlation analysis, we found clear negative correlation between the amounts of amyloid in culture fluids with binding to C3b (Figure 4A) or with the frequencies of opsonophagocytosis by PMNs (Figure 4B). In addition, amounts of amyloid in the culture fluids showed strong negative correlation with SAP binding (Figure 4C) but did not correlate with amounts of C1q binding (Figure 4D). No significant correlation between amyloid production and binding to C4BP was found (Pearson’s correlation; *r*: −0.0838, *p*: 0.820), but a significant negative correlation between amyloid production with FH binding was detected for a reduced proportion of the strains (*r*: −0.3634; *p* < 0.02). Therefore, *S. sanguinis* strains with increased production of extracellular amyloid components are less susceptible to SAP binding, C3b deposition and opsonophagocytosis by PMNs.

### 2.5. Deletion of srtA Impairs Amyloid Formation during Different Forms of S. sanguinis Growth

**Figure 5:**
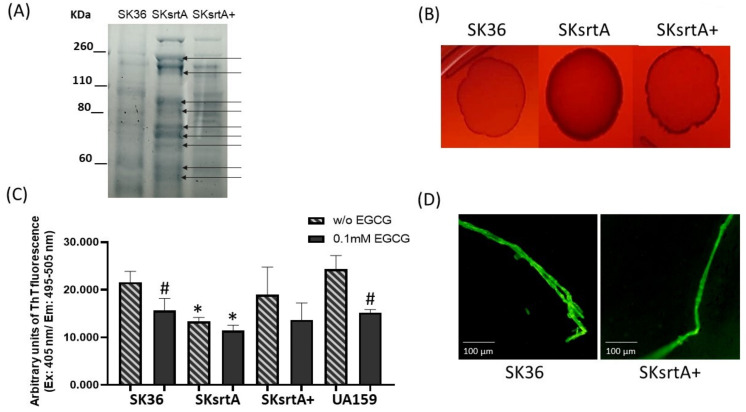
Effects of *srtA* deletion on CR-binding and amyloid production. (**A**) Protein band profiles of samples of culture supernatants obtained from SK36, SKsrtA and SKsrtA+. Equivalent amounts of protein (10 µg/lane) were resolved in 8% SDS-PAGE gels and stained with Coomassie blue. Arrows indicate band proteins detected in SKsrtA but not in SK36 and SKsrtA+. (**B**) Macrocolonies of SK36, SKsrtA and SKsrtA+ formed over 72 h on BHI agar supplemented with CR. (**C**) Samples with equivalent amounts of protein (1 µg/mL) of concentrated culture supernatant were incubated (4 °C; 48 h) with 0.1 mM EGCG or PBS (without EGCG), ThT was added and the fluorescent intensities were measured in a GloMax^®®^ Discover System (AFC filter; excitation: 405 nm; emission: 495–505 nm). Columns represent means of data obtained from three independent cultures; bars indicate standard deviation. Asterisks indicate significant differences in relation to SK36 under the same condition; hashtags indicate significant differences in relation to samples without EGCG obtained from the same strain (Kruskal–Wallis with Dunn’s post hoc test; *p* < 0.05). (**D**) Representative fluorescence microscopy images of fibrous aggregates detected in samples of culture supernatants stained with ThT obtained from SK36 and SKsrtA+.

The sortase A protein targets expressed by *S. mutans* (P1 and WapA) and *Streptomyces coelicolor* (chaplins) are amyloidogenic proteins [27,30,31]. Moreover, in *S. mutans*, sortase A expression is required for amyloid polymerization during biofilm growth [23]. Thus, we assessed the effect of sortase A gene (*srtA*) deletion in SK36 on amyloid production phenotypes under different forms of bacterial growth (planktonic, growth in solid media and biofilm). Initially, we monitored the protein band profiles of cell-fee samples of culture supernatants in the *srtA* mutant (SKsrtA), parent strain (SK36) and complemented mutant (SKsrtA+) using 8% SDS-PAGE gels. As expected, the SKsrtA samples showed a more complex band profile when compared to SK36 and SKsrtA+, indicating an increased number of proteins being released into the culture medium (Figure 5A). We next compared the CR-binding phenotypes of macrocolonies formed by these strains over 72 h of incubation in BHI agar supplemented with CR. As shown in Figure 5B, the SKsrtA macrocolony showed increased binding to CR, when compared to macrocolonies formed by SK36, whereas the CR-binding phenotype was restored in SKsrtA+ (Figure 5B). We then quantified amounts of amyloid-like components in concentrated samples of culture supernatants with equivalent amounts of protein (1 µg/mL), after incubation (4 °C; 48 h) with 0.1 mM EGCG (or PBS), using ThT and fluorimetry (Figure 5C). The results from three independent cultures obtained per strain are shown in Figure 5C, and they indicate that culture fluids of SKsrtA had significantly lower levels of fluorescent components than supernatants of SK36. Amounts of fluorescent components were restored in SKsrtA+, although in larger variations when compared to SK36, which could reflect the instability of plasmid pDL278::*srtA* in cultures not supplemented with spectinomycin, the antibiotic for maintaining plasmids (Figure 5C). Finally, EGCG significantly inhibited amyloid production in SK36, although it had limited effects on samples of SKsrtA and SKsrtA+ (Figure 5C). As expected, EGCG significantly inhibited amyloid production in culture fluids of the reference *S. mutans* strain UA159 (Figure 5C). Amyloid-like components detected in ThT-stained samples of culture supernatant were also evaluated via fluorescence microscopy (Figure 5D). Compatible with the fluorimetric quantitative analysis, samples of SK36 and SKsrtA+ showed fluorescent fibrous aggregates (Figure 5D), which were infrequent in SKsrtA samples and not detected BSA control samples. Therefore, *srtA* deletion influences *S. sanguinis* binding to CR and reduces the formation of amyloid-like components in culture fluids.

**Figure 6:**
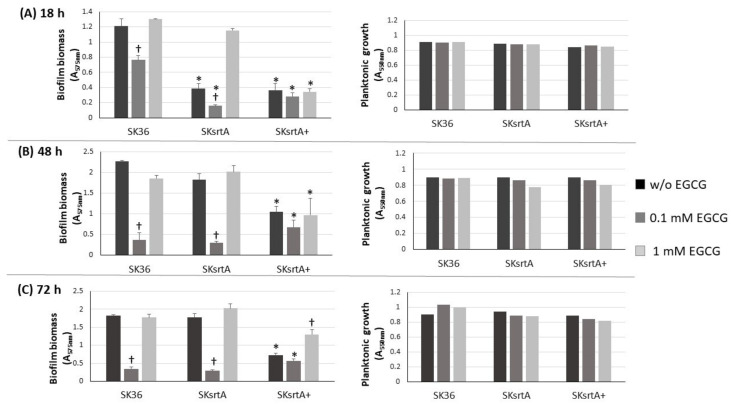
Effect of *srtA* deletion on biofilm formation in the presence or absence of EGCG. Biofilms were formed on 96-well plates in 1% sucrose BHI supplemented or not with EGCG (0.1 or 1 mM) over 18 (**A**), 48 (**B**) and 72 h (**C**) of incubation (indicated in horizontal panels). The absorbances (A_757 nm_) of ethanol eluates of the biofilms previously stained with crystal violet were used as measures of biofilm biomass. Planktonic growth of the same cultures (A_550 nm_) monitored at the respective incubation periods are shown on the right (**A**–**C**). Columns represent means of triplicates of one representative experiment; bars indicate standard deviations. Asterisks indicate significant difference in relation to SK36 under the same conditions; crosses indicate significant differences in relation to the same strain grown in medium without EGCG (Kruskal–Wallis with Dunn’s post hoc test; *p* < 0.05).

We also investigated the effect of *srtA* deletion on biofilm formation in the presence and absence of EGCG by comparing the biomass of biofilms formed in 96-well plates in 1% sucrose BHI with or without EGCG (at 0.1 and 1 mM) in the same assays used for comparisons of clinical strains (Figure 3). As shown in Figure 6, the SKsrtA mutant showed reduced biofilm formation either in medium with or in medium without 0.1 mM EGCG when compared to SK36 grown under the same conditions over 18 h (upper panel). However, significant differences in biofilm biomass between SKsrtA and SK36 could not be detected after 48 and 72 h of growth (Figure 6, lower panels). The addition of 0.1 mM EGCG to BHI further reduced biofilm formation in SKsrtA as compared to BHI without EGCG, whereas EGCG at 1.0 mM did not influence the biofilm formation of SKsrtA (Figure 6), as observed for other strains (Figure 3). Biofilm phenotype was not restored in SKsrtA+, which again could be due to the instability of plasmid pDL278::*srtA* during biofilm maturation. As expected, EGCG did not influence the planktonic growth of the tested strains (Figure 6, right panels). Because differences in amyloid production could be masked by other exopolymers (including eDNA and exopolysaccharides) and/or by BHI tissue components, which would bind at different extents to the tested strains, it was thus difficult to identify amyloid contribution to biofilm biomass phenotypes of SK36, SKsrtA and SKsrtA+ in 1% sucrose BHI.

**Figure 7:**
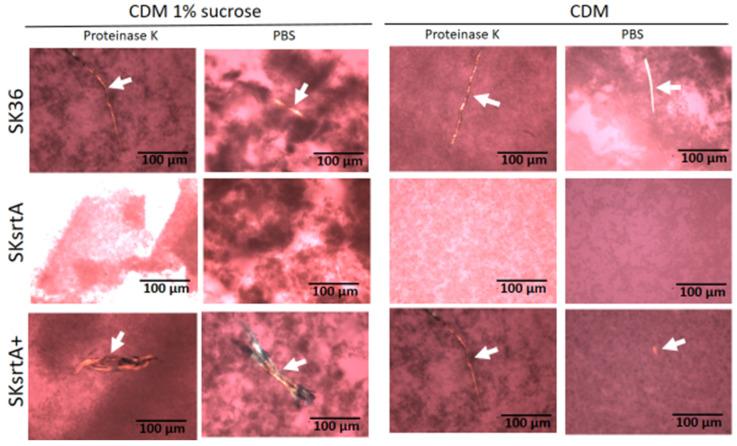
Effect of *srtA* deletion on amyloid fibril production in biofilms. Biofilms formed by strains SK36, SKsrtA and SKsrtA+ in 1% sucrose CDM or in CDM without sucrose over 72 h, were collected, treated with proteinase K (or PBS), stained with CR and analyzed under cross-polarized light. Digital images were obtained at 200× magnification. Arrows indicate birefringent fibrils. Strain identities are indicated to the left of the horizontal panels.

To circumvent the potential influence of BHI components and sucrose-derived glucan in the assessment of amyloid production in biofilms formed by SKsrtA, SK36 and SKsrtA+, we next investigated the production of amyloid components in biofilms formed over 72 h in CDM supplemented or not with 1% sucrose (in 24-well plates). Biofilms were then treated with proteinase K (or PBS as control) and stained with CR for cross-polarized light microscopy to allow amyloid detection. As shown in Figure 7, the SK36 and the complemented strain SKsrtA+ formed biofilms containing birefringent amyloid fibrous aggregates, which were resistant to proteinase K, as observed by CR staining and cross-polarized light microscopy. These fibrils were formed either in the presence or absence of sucrose, indicating that glucan does not impair the assembly of amyloid fibrils. Importantly, amyloid aggregates could not be detected in biofilms formed by SKsrtA (Figure 7). To further confirm the amyloid nature of the fibrous aggregates detected in CR-stained biofilms, we analyzed the binding of these component to ThT. Thus, samples of biofilms formed by the same strains (and under the same conditions used for CR staining) were treated with proteinase K, stained with ThT, and analyzed via fluorescence microscopy. As shown in Figure S1, fluorescent aggregates of similar morphology of those detected via cross-polarized light microscopy (Figure 7) were detected in biofilms formed by SK36 and SKsrtA+ but not in biofilms formed by the SKsrtA mutant. Therefore, *S. sanguinis* produces amyloid aggregates during biofilm formation, in a fashion dependent on *srtA* expression. 

**Figure 8:**
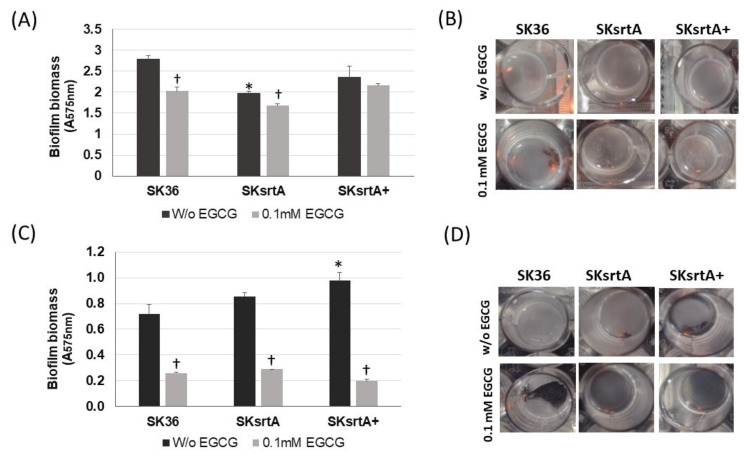
Effects of *srtA* deletion in *S. sanguinis* biofilm phenotypes. Biofilms were formed over 18 h by strains SKsrtA, SK36 and SKsrtA+ in 24-well microtiter plates with CDM (without sucrose) or 1% sucrose CDM, in the presence or absence of 0.1 mM EGCG. Biofilms were gently washed and stained with crystal violet. The absorbances of ethanol eluates of the stained biofilms (A_575 nm_) were used as measures of biofilm biomass. (**A**) Biomass of biofilms formed in CDM. (**B**) Images of biofilms formed in CDM. (**C**) Biomass of biofilms formed in 1% sucrose CDM. (**D**) Images of biofilms formed in 1% sucrose CDM. Asterisk indicates significant difference in relation to SK36 under the same conditions; cross indicates significant difference in relation to biofilm formed in the absence of EGCG by the same strain (Kruskal–Wallis with Dunn’s post hoc test).

To explore the effects of amyloid on the stability of biofilms, we next quantified biofilms formed under the same conditions in the presence and absence of 0.1 mM EGCG. As shown in Figure 8A,B, biofilms formed by SKsrtA in CDM (without sucrose) were less stable and more easily detached during the gentle washing steps, when compared to biofilms formed by SK36 or SKsrtA+ under the same conditions. Moreover, EGCG reduced the stability of SK36 biofilms, but had limited effects on the stability of biofilms formed by SKsrtA. Supplementation of CDM with 1% sucrose increased the biofilm biomass in all strains regardless of the addition of EGCG, as observed by visual inspection of the 24-well plates (Figure 8D). However, these increases could not be precisely quantified (Figure 8C), because these robust biofilms more easily detached from the 24-well plates during the washing steps, especially when formed in the presence of EGCG (Figure 8D). Of note, when visually compared before the washing steps, the biofilms formed by SKsrtA in 1% sucrose CDM appeared to be of reduced biomass as compared to SK36 in the same medium.

### 2.6. Deletion of srtA Increases S. sanguinis Susceptibility to C3b Deposition, Affects Its Interaction with PMNs and Impairs Persistence in Human Blood

**Figure 9:**
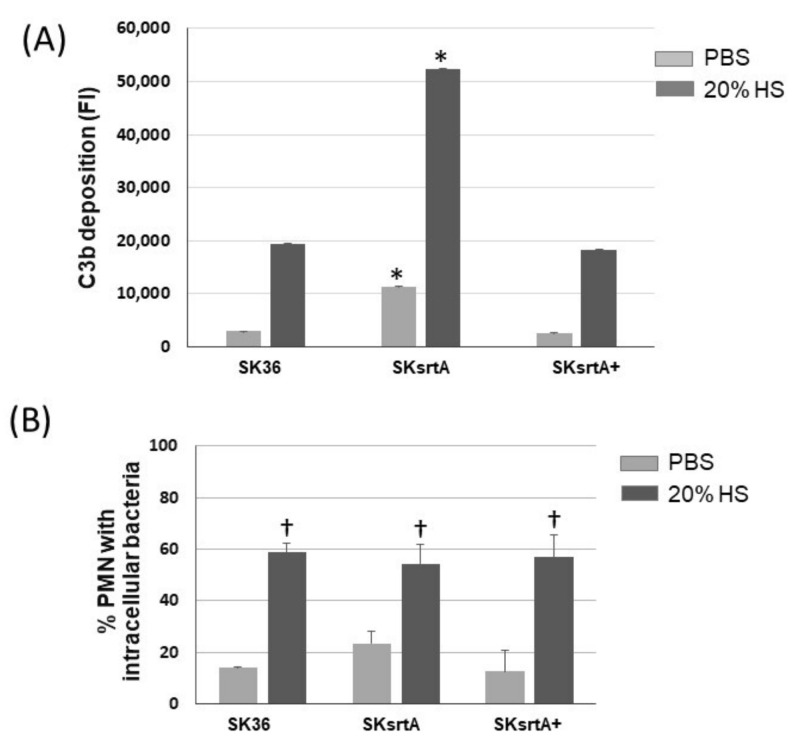
Deposition of C3b and opsonophagocytosis by human PMNs in *S. sanguinis* strains. (**A**) The intensities of C3b deposition (FI) on strains SK36, SKsrtA and SKsrtA+ (at A_550 nm_ 0.3) treated with 20% human serum (HS) or PBS (negative control) were determined using flow cytometry. Columns represent the means of triplicates of one representative experiment; bars indicate standard deviations. Asterisks indicate significant differences in relation to SK36 under the same conditions (Kruskal–Wallis with Dunn’s post hoc test; *p* < 0.05). (**B**) Frequencies of bacterial phagocytosis by PMNs isolated from peripheral blood determined via flow cytometry. PMNs were exposed to FITC-labeled bacteria in the presence of PBS or 20% HS. Columns represent means of data obtained in three independent experiments; bars indicate standard deviation. No significant differences were observed between strains under the two conditions tested; cross indicates significant difference (*p* < 0.05) in relation to the same strain in PBS (Kruskal–Wallis with Dunn’s post hoc test).

Because there was a negative correlation between the amounts of amyloid-like compounds in culture fluids and C3b deposition in the set of nine *S. sanguinis* strains (Figure 4), we further assessed the effects of *srtA* deletion in the susceptibility of SK36 to C3b deposition and opsonophagocytosis by PMNs. As shown in Figure 9A, there was a significant increase in C3b deposition on the surface of the SKsrtA mutant treated with human serum (HS) as compared to SK36. In addition, the C3b deposition phenotype was fully restored in the SKsrtA+. Unexpectedly, no increase in the frequency of opsonophagocytosis was detected in the SKsrtA as compared to SK36 in our flow cytometry assays (Figure 9B). Although SKsrtA formed longer chains when compared to SK36 in overnight cultures (18 h), no significant differences in chain length could be detected between these strains when at the growth phase used for phenotypic comparisons (A_550 nm_ 0.3). 

**Figure 10:**
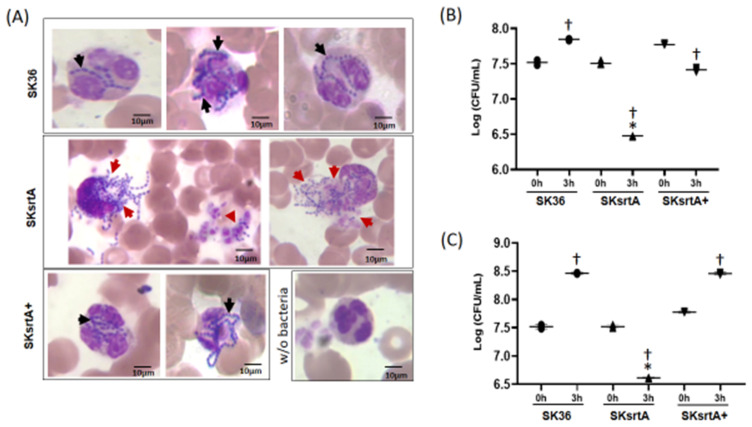
Effects of *srtA* on *S. sanguinis* interactions with PMNs and persistence in human blood. (**A**) The strains SK36, SKsrtA and SKsrtA+ (at A_550 nm_ 0.3) were harvested, suspended in human blood and incubated (37 °C) for 5 min. Bacterial interactions with PMNs were then analyzed via light microscopy in samples stained with May–Grunwald Giemsa stain. Digital images were obtained at 1000× magnification. Strains SK36 and SKsrtA+ were clearly associated with intact PMNs (black arrows), whereas PMNs associated with SKsrtA typically showed release of intracellular content suggestive of degranulation and/or NETosis (red arrows). (**B**,**C**) Bacterial survival in human blood. Equivalent number of strains (at A_550 nm_ 0.3) were suspended in fresh blood and incubated (37 °C; 3 h) under orbital rotation (**B**) or statically (**C**). The numbers of viable bacteria (log CFU/mL) were determined in BHI agar plates just after bacterial suspension in blood (0 h) and after 3 h of incubation. Asterisks indicate significant difference in relation to SK36 at the same time point; cross indicates significant difference in relation to t0 h for the same strain (ANOVA with Tukey’s post hoc test; *p* < 0.05).

To further investigate the killing of *S. sanguinis* by PMNs mediated by serum/blood components, we analyzed Giemsa-stained samples of SK36, SKsrtA and SKsrtA+ incubated with fresh peripheral blood, using light microscopy. As shown in Figure 10A, unlike the SK36 chains, which could be clearly observed inside PMNs, the SKsrtA were frequently associated with a diffuse material apparently resulted from the release of PMNs intra-cellular content, suggesting the production of neutrophil extracellular traps (NETs). In addition, PMNs challenged with SKsrtA+ showed phenotypes similar to those observed in PMNs exposed to SK36. The SKsrtA mutant also showed reduced capacity to persist in human blood after 3 h of incubation under orbital rotation (for increased contact with PMNs) when compared to SK36 and SKsrtA+ (Figure 10B). In addition, while SK36 was able to grow in blood suspensions under static incubation, the SKsrtA showed a significant reduction in cell counts under this condition (Figure 10C). Moreover, the blood persistence phenotype was rescued in the complemented strain (Figure 10C). Thus, *srtA* deletion increases *S. sanguinis* susceptibility to complement-mediated immunity, affects its interactions with PMNs and reduces its persistence in human blood. 

## 3. Discussion

The roles of *S. sanguinis* as a key beneficial organism of dental biofilms and important cardiovascular pathogen emphasize the need for deciphering the mechanisms through which this species forms biofilms and copes with host immune functions present in the oral cavity and in extra-oral tissues. A high diversity of phenotypes associated with biofilm formation, host persistence and cardiovascular infection has been reported among strains [17,32], and might reflect strain-specific capacities to produce and interact with exopolymers modulating these processes [17]. Although functional amyloid is a ubiquitous class of bacterial exopolymers involved in biofilm formation and host immune evasion [19,20], there are no studies addressing the roles of these exopolymers in *S. sanguinis* biology. Here, we show that amyloid-like aggregates are produced by several *S. sanguinis* strains and provide evidence that these polymers are linked to biofilm formation and to evasion to the complement system, a major branch of immune surveillance of the cardiovascular system. Moreover, we show that *S. sanguinis* relies on the expression of sortase A to produce amyloid exopolymers in biofilms, implying the contribution of sortase A and as yet unidentified sortase A substrates in amyloidogenesis. 

Surface-associated functional amyloid generally contributes to bacterial adhesion and biofilm formation through its interaction with eDNA and exopolysaccharides [33,34,35,36]. The production of eDNA by *S. sanguinis* is required for bacterial aggregation and biofilm initiation [14,15], whereas the presence of sucrose, the unique substrate for the synthesis of glucan, is also required for biofilm maturation [7,15,37]. Glucan produced by *S. sanguinis* shows, however, lower stability than glucan produced by the competitor cariogenic species, *S. mutans* [12,16,17], which explains the prominent role of sucrose on *S. mutans* virulence, as compared to other commensal streptococci, in dental communities [38]. Thus, biofilms formed by *S. sanguinis* likely have shorter lifecycles when coexisting in homeostasis with host immune functions. Our findings indicate that independently of the presence of sucrose-derived glucan, *S. sanguinis* produces fibrous aggregates with multiple traits typical of amyloid, including protease resistance, CR-promoted birefringence under cross-polarized light and binding to ThT. These aggregates are also quite similar to amyloid polymers reported in *S. mutans* biofilms [23,25], which are further produced in a fashion dependent on sortase A expression [23,28].

Normally, amyloid aggregates are formed by multiple amyloid fibrils (with 5 to 20 nm in width) composed by one to multiple laterally associated protofilaments, each consisting of self-assembled stacks of monomeric peptide subunits polymerized in cross-β structures (β-strands stacked perpendicularly to the filament longer axis) [39]. Purified fragments of protein substrates of sortase A expressed by *S. mutans* are assembled into amyloid fibrils under certain in vitro conditions [23,28]. These include P1 (also known as SpaP, AgI/II or Pac) and WapA [23,28]. However, full-length P1 or WapA or the natural fragments of these proteins fail to form detectable amyloid components during biofilm formation in a *S. mutans srtA* deletion mutant [23,28]. A hypothesis to explain these findings suggested that peptideoglycan-anchored forms of P1 and WapA would be necessary as nucleator proteins for triggering the polymerization of additional monomers (amyloid fibril elongation) on the bacterial surface [23,28]. This process would be analogous to the nucleation-precipitation mechanism established for curli amyloids produced in vivo by *E. coli* [40]. Our findings in *S. sanguinis* are compatible with previous studies in *S. mutans* [23,28] and strengthens the need for studies clarifying the function of sortase A in the biogenesis of surface-associated amyloid polymers in *S. sanguinis*, as well as in other Gram-positive bacteria, during biofilm formation.

Besides P1 and WapA polypeptides, *S. mutans* expresses another two amyloidogenic proteins, the secreted protein Smu.63 and the serotype-restricted collagen- and laminin-binding protein Cnm, which are, respectively, required for sucrose-independent biofilm formation [23,28,41] and cardiovascular virulence [25]. Using BLASTp analysis, we confirmed that *S. sanguinis* genomes harbor genes encoding for P1 homologues (52% identity with *S. mutans* P1) but not for WapA, Smu63c or Cnm. However, whereas the *S. mutans* genome harbors only six genes encoding proteins with an LPxTG motif [42], a much larger number of sortase A substrates (thirty-three) was identified in the *S. sanguinis* SK36 genome [8]. Therefore, future studies are needed to identify which of these substrates are involved in amyloid production by *S. sanguinis*; purified proteins could be applied in the analyses of the hierarchical structure of these polymers and kinetics of amyloid aggregation.

The fibrilization of the C-terminal fragment of *S. mutans* P1 is inhibited by EGCG at 0.1 mM. Compatible with this effect, this flavonoid inhibits sucrose-independent biofilm formation in wild-type strain but not in P1-deficient mutant [28]. EGCG interferes with the amyloid fibrilization of multiple proteins [43,44,45,46], affecting not only the initial phases of amyloid polymerization but disassembling pre-formed amyloid fibers into smaller aggregates [47]. The anti-amyloidogenic effects of EGCG might therefore be detected in different strains, despite potential differences in the expression of amyloidogenic proteins, which would influence on kinetics of amyloid polymerization. Our findings that 0.1 mM EGCG inhibits biofilm formation in several *S. sanguinis* strains is thus compatible with the hypothesis that the P1 homologue and/or other sortase A substrates expressed by *S. sanguinis* contribute to amyloidogenesis. Apart from its anti-amyloidogenic properties, EGCG also modulates additional bacterial functions at different concentration ranges, including cell wall synthesis, the activity of metabolic enzymes, the synthesis of DNA and fatty acids and quorum sensing [45], which might explain the inverse effects of EGCG on the biofilm maturation of the *S. sanguinis* when at 1 mM.

Whether *S. sanguinis* amyloids also contribute to cardiac vegetations and/or atherogenesis needs to be addressed, because human and bacterial amyloid fibrils can bind to plasminogen, fibronectin and laminin [48,49,50,51]. Moreover, SAP (and/or C1q) binding to amyloid modulates inflammation and tissue damage in multiple diseases, including cardiovascular, Alzheimer’s disease, systemic amyloidosis and systemic candidiasis [52,53,54,55]. SAP (also known as pentraxin 2) is abundant in blood and binds to C1q, promoting the activation of the classical pathway of the complement in streptococci and other microorganisms [56,57]. Here, we showed a strong negative association between the amounts of amyloid-like components in the culture fluids of *S. sanguinis* strains, with the intensities of strain binding to SAP, C1q and C3b having been previously reported [8]. We thus speculate that extracellular amyloid could sequester the complement activating proteins (SAP and C1q), avoiding complement activation on the bacterial cell wall. 

Consistent with our findings, the production of curli amyloid fibrils promotes the evasion of the classical pathway of complement activation in *Escherichia coli* [32]. Interestingly, two strains (SK353 and SK678) producing reduced levels of amyloid associated with high-binding levels to SAP are also defective in expressing *pepO* [58], the gene encoding for the metallo-endopeptidase PepO required for complement evasion in *S. mutans* and *S. sanguinis* [58,59], as well as in other pathogenic streptococci [60,61]. It is of note that PepO is part of the M13 family of zinc metallo-endopeptidases, which includes mammalian peptidases capable of degrading amyloid beta peptides [62]. Further studies will be thus necessary to investigate whether PepO modulates amyloid fibril polymerization in *S. sanguinis*. 

Strengthening the associations between amyloid production with reduced binding to SAP/C1q/C3b in wild type strains, the SKsrtA mutant showed a significant increase in binding to C3b, which would ultimately account for reduced systemic virulence. Unexpectedly, the increased C3b deposition on SKsrtA did not reflect on its increased interaction with PMNs measured in flow cytometry assays, despite the major role of C3b in *S. sanguinis* opsonization [9,17]. Similarly, another *S. sanguinis srtA* mutant obtained in strain DSS-10 showed a modest increase in opsonophagocytic rates, despite its increased sensitivity to blood-mediated killing [63]. Because effector molecules generated by complement activation on bacterial species, e.g., C3a, C5a and CAM, stimulate PMN activation and NETosis [64,65,66], we speculate that the increased complement activation on SKsrtA enhanced bacterial killing and NETosis by PMNs, compromising the flow cytometry detection of PMNs associated with SKsrtA. This hypothesis was consistent with the microscopy analysis of PMNs challenged with SKsrtA, SK36 and SKsrtA+. Further studies will be performed to confirm the SKsrtA induction of NETosis and the potential participation of the complement system in this process.

The reduced persistence of SKsrtA in human blood as compared to SK36 further indicates an increased sensitivity of SKsrtA to blood antimicrobial components (either derived from PMNs or not) and/or defects in blood fitness, as reported for *srtA* mutants obtained in *Streptococcus pyogenes* [67]. Interestingly, another *srtA* mutant obtained in SK36 showed only a two-fold reduction in competitiveness in a competitive rabbit model of IE [8]. Moreover, the screening of third one single mutants obtained in SK36 which were defective in sortase A protein substrates could not identify individual genes contributing to virulence in this same model [8]. Apart from these issues, our findings that *srtA* deletion promotes defects in amyloid production, affecting complement activation, interaction with human PMNs and persistence in blood, highlight the complexity of *srtA* roles in the *S. sanguinis* capacities to evade and to modulate host immunity in the bloodstream and/or in infected tissues.

In summary, here we described the production of amyloid fibrils by *S. sanguinis* strains and provided evidence that these exopolymers contribute to strain-specific phenotypes of biofilm formation and complement evasion. We further provided evidence that the deletion of *srtA* impairs amyloid production by *S. sanguinis*, affecting its capacity to form biofilm in the absence of sucrose and to evade blood-mediated immunity. These findings highlight the need for identifying amyloidogenic cell wall-anchored proteins of *S. sanguinis*, a topic of our future studies.

## 4. Materials and Methods

### 4.1. Strains, Culture and Staining Conditions

Strains used in this study are described in Table 1 [7,68,69]. Streptococcal strains were routinely grown in brain heart infusion (BHI) agar (BD Difco, Franklin Lakes, NJ, USA) at 37 °C in an aerobic atmosphere with 10% CO_2_ in air. When needed, growth media were supplemented with appropriate antibiotics (erythromycin (10 μg/mL) or spectinomycin (200 μg/mL) (Merck Labs, Darmstadt, Germany). For phenotypic analyses, overnight cultures with adjusted absorbances were prepared in BHI, diluted 1:100 into fresh BHI or chemically defined medium (CDM) [70] supplemented or not supplemented with 1% sucrose. For some experiments, medium was also supplemented with freshly prepared solution of 20 mM epigallocatechin-3-gallate (EGCG) (Sigma-Aldrich, St. Louis, MO, USA) in dH_2_O, which were filtered through polyethersulfone filters with pores of 0.22 µm in diameter (Kasvi K18-230). *Escherichia coli* was grown in a 37 °C shaker incubator in Luria–Bertani broth (BD Difco, USA) supplemented with ampicillin (100 µg/mL). Solutions of CR (Sigma-Aldrich) and thioflavin T (ThT; Sigma-Aldrich) were prepared, as previously described [23] and according to the supplier. Briefly, 80 mL of CR solution (0.5 g in 80 mL of 100% ethanol) were mixed with 20 mL of NaCl solution (2.0 g in 20 mL of dH_2_O) and filtered using polyethersulfone membranes with pores of 0.22 µm in diameter; Kasvi). ThT 20 mM stock solution was prepared by dissolving 0.0064 g of ThT (Sigma-Aldrich) in 1 mL of phosphate buffer (10 mM Na_2_HPO_4_; 150 mM NaCl; pH 7.0).

### 4.2. Construction of the srtA Isogenic Mutants and Complemented Strain

The *srtA* nonpolar deletion mutant was obtained in SK36 using a PCR-ligation strategy, as previously described [71]. Primers used for the construction of mutant and complement strains and for RT-qPCR analysis are shown in Table 2. Briefly, a recombinant allele (2245 bp) was constructed by replacing the *srtA* gene (SSA_1219; 756 bp) with an erythromycin resistance cassette (Erm^r^) obtained from plasmid pVA838 [72]. The recombinant allele was transformed into SK36 to generate the mutant strain SKsrtA, which was placed on BHI agar supplemented with erythromycin. The SKsrtA was confirmed via PCR and DNA sequencing analysis. The *srtA*-complemented strain (SKsrtA+) was obtained by transforming SKsrtA with plasmid pDL278::*srtA* harboring a spectinomycin resistance gene [73] and a full-length copy of *srtA* with its promoter region (1529 pb), which was amplified from the SK36 genomic DNA with primers C1 and C2 (Table 2). RT-qPCR analysis was performed, as described in previous studies [59], to confirm *srtA* transcription in SKsrtA+, using specific primers for *srtA* and 16S rRNA gene as reference (Table 2).

### 4.3. Detection of Amyloid Fibrils in Biofilms by Cross-Polarized Light Microscopy and Fluorescence Microscopy

Amyloid fibrils in mature biofilms were analyzed as described elsewhere [27], with some modifications. Briefly, bacterial strains were grown in CDM (37 °C, 10% CO_2_ in air for 18 h) and these cultures were diluted into fresh CDM with and without 1% sucrose to an adjusted absorbance (A_550 nm_ 0.03). The culture dilutions were then transferred to 24-well plates (Kasvi) (1.5 mL/well), which were incubated for 72 h under the same conditions. Afterwards, the biofilms were suspended in their culture fluids and these samples were harvested (6000× *g*, 3 min., 4 °C). The biofilm pellets were then suspended in a proteinase K solution (10 µg/uL; ThermoFisher) or PBS (negative control), incubated for 3 h at 37 °C and the enzyme reaction was halted by adding phenylmethylsulfonyl fluoride (PMSF; 2 mM) [27]. The biofilm samples were harvested again (6000× *g*; 3 min.; 4 °C) and suspended in 100 µL of 0.5% CR solution. Aliquots of these suspensions (20 µL) were transferred to glass slides and analyzed using a polarized light microscope (Leica DMLP). Birefringence was detected by applying the polarizer at 90°. Digital images were obtained under polarized light at 200× total magnification. Alternatively, the harvested samples of biofilms were suspended in 500 µL of PBS with 2.5 uM ThT and transferred to glass slides for analysis in a fluorescence microscope equipped with a digital camera (Zeiss Axiovert 40 CFL; filter set 09-Ex BP 450–490; Em LP 515). Digital images were then obtained at 200× total magnification.

### 4.4. Macrocolony Phenotype

The macrocolony morphology of strains was analyzed as previously described [34] with modifications. Briefly, strains were grown in BHI for 18 h (37 °C; 10% CO_2_in air), and aliquots of 10 µL of these cultures with adjusted absorbance were spotted on BHI agar supplemented with CR (40 µg/mL) and incubated under the same conditions for 1 to 3 days. Digital images of macrocolony morphologies were obtained each day. All the strains were placed in the same agar plate for comparison.

### 4.5. Preparation of Culture Supernatants

Amyloid formation was assessed in samples of concentrated supernatants of *S. sanguinis* cultures obtained in sucrose-free CDM with or without EGCG. The samples were prepared as previously described [74] with minor modifications. Briefly, aliquots of 18 h cultures in CDM [70] with an adjusted numbers of cells were diluted (1:10) into 40 mL of fresh CDM and incubated for 18 h (37 °C; 10% CO_2_ in air). The A_550 nm_ of the cultures were monitored, and 50 mL of the cultures centrifuged twice (6000× *g*; 4 °C; 4 min.) for collection of culture supernatants. Samples were then filtered through polyethersulfone membranes (0.22 µm-pore diameter; Kasvi) for the removal of remaining cells. The pH of these culture fluids were then neutralized by adding 1 M NaOH, and phenylmethylsulfonyl fluoride (PMSF) was added to a final concentration of 10 µM for protease inhibition. The samples were then dialyzed for 18 h at 4 °C against cold phosphate buffer (Na_2_HPO_4_; NaH_2_HPO_4_; 0.2 M; pH 6.5) and then against cold 1:100 diluted Tris-HCl buffer (0.125 M; pH 6.8). Afterwards, the samples were 100-fold concentrated by freeze-drying. The protein concentration of samples were then determined using a Bradford assay (BioRad, Darmstadt, Germany) and the integrity of protein samples monitored in 8% SDS-PAGE gels stained with Coomassie blue.

### 4.6. Quantitative Analysis of Amyloid through Spectrofluorimetry

To assess the amounts of amyloid fibrils in culture supernatants, samples of concentrated culture fluids (1 µg/mL diluted in 50 mM NaH_2_PO_4_, 300 mM NaCl; pH 8.0) and 2.5 µM of ThT were added and incubated at rt for 30 min. The samples were then transferred to black polystyrene 96-well microtiter plates (Corning, New York, NY, USA) (100 µL/well in quadruplicates) and the intensities of fluorescence were determined using a spectrofluorometer (Promega GloMax^®®^ Discover System, Madison, WI, USA) at 425 nm excitation wavelength and emission spectra collected at 495 to 505 nm. Serial dilutions of BSA were used as negative control. A standard curve of the concentrated culture supernatant of *S. mutans* UA159 (0.125; 0.5; 0.75; 1; 1.25; 1.5 and 2 µg/µL) was also used as reference in all the experiments to ensure ThT fluorescence under a linear range of detection. Similar assays were performed with strains SKsrtA, SKsrtA+ and SK36 using samples of similarly prepared culture fluids obtained in CDM supplemented or not with 0.1 mM EGCG and stirred for 24 h at 4 °C. Samples were collected from cultures obtained on three different days for each strain analyzed. To monitor the presence of amyloid-like aggregates, aliquots of ThT-stained samples were also analyzed using fluorescence microscopy as described above.

### 4.7. Biofilm Formation Assays

Biofilm formation in the presence or absence of EGCG was analyzed using 96-well microtiter plate assays as previously described [17] with minor modifications. Briefly, 18 h BHI cultures with adjusted absorbances were diluted (1:10) in fresh 1% sucrose BHI or 1% sucrose BHI supplemented with EGCG at 0.1 and 1 mM [23]. Aliquots of the culture dilutions were then transferred to polystyrene 96-well plates (CralPlast) (200 µL/well), which were then incubated (37 °C, aerobiosis under gentle agitation-80 rpm) for 18 h. Afterwards, biofilm fluids were removed, and the plates were gently washed twice via immersion in water. Biofilms were then stained with 1% crystal violet for 30 min (rt), washed twice with water and dried for 1 to 2 h at rt. Then, the stain was eluted from biofilms via incubation with ethanol (200 µL/well; 30 min, rt), and the absorbances (A_575 nm_) of the eluates were expressed as indirect measures of biofilm biomass. The planktonic growth of strains was monitored in the same culture dilutions, which were incubated under similar conditions. Three independent experiments were performed in triplicate.

Biofilms were also formed in 24-well plates using cultures in CDM and in 1% sucrose CDM, which were supplemented or not supplemented with EGCG. Briefly, 18 h cultures in CDM were diluted (to A_550 nm_ 0.03) into fresh CDM or 1% sucrose CDM (with and without 0.1 mM EGCG) and transferred to 24-well polystyrene plates (Kasvi). After incubation (37 °C; 10% CO_2_) for 72 h, the biofilm fluids were carefully removed by pipetting and gently washed by pipetting with distilled water. The biofilms were then stained with 1% crystal violet and processed, as previously described for quantification of biofilm biomass.

### 4.8. C3b Deposition

The deposition of C3b on *S. sanguinis* strains was determined as previously described [17]. Briefly, approximately 10^7^ CFU of bacteria collected from culture at mid-log phase (A_550 nm_ 0.3) via centrifugation (10,000× *g*, 4 °C) were washed twice with PBS (pH 7.4), resuspended in 20 µL of 20% serum in PBS and incubated at 37 °C for 30 min. Cells were then washed twice with PBS Tween 0.05% (PBST) and incubated on ice with fluorescein isothiocyanate (FITC)-conjugated polyclonal goat anti-human C3 IgG antibody (ICN, Seattle, CA, USA) (1:300 in PBST). Afterwards, cells were washed twice in PBST and fixed in 3% paraformaldehyde for flow cytometry analysis using a FACSCalibur flow cytometer (BD Biosciences). At least 25,000 bacteria were gated using forward- and side-scatter parameters for the determination of the geometric mean of fluorescent intensity (MFI) in positive cells. The intensities of C3b deposition were expressed using a fluorescent index (FI), which was calculated by multiplying the MFI values by the percentage of C3b-positive cells. Bacterial cells treated only with PBS were used as negative control in all the assays. Bacterial strains treated with heat-inactivated serum (56 °C for 20 min) were also used in preliminary experiments as negative controls to confirm that non-complement serum components had irrelevant effects on strain comparisons.

### 4.9. PMN Isolation and Phagocytosis Assay

Human PMNs were isolated from fresh heparinized blood collected from a healthy volunteer under the approval of the Ethics Committee of the Piracicaba Dental School, State University of Campinas (CEP/FOP-UNICAMP; CONEP protocol n^o^. 58699622.4.0000.5418). PMNs were isolated using double gradient (1119 and 1083 density Histopaque; Sigma-Aldrich) followed by the removal of red blood cells via hypotonic lysis. The isolated PMNs were suspended in RPMI 1640 medium (GIBCO, Life Technologies, Waltham, NY, USA) supplemented with inactivated 10% fetal bovine serum. Cell viability (>98%) and purity (>95%) were monitored using trypan blue exclusion and May–Grunwald Giemsa staining, respectively. For the phagocytosis assay, bacteria were labeled with FITC as previously described [17]. Briefly, bacteria were harvested via centrifugation from cultures at A_550 nm_ 0.3, washed twice with PBS, suspended in FITC (Sigma) solution (1.7 mg/mL in carbonate buffer (Na_2_CO_3_ 0.15 M, 0.9% NaCl; pH 9) and incubated at rt in the dark for 1 h. Bacteria were then collected via centrifugation, washed three times with PBST for application in the phagocytosis assays or stored overnight in 10% glycerol at −70 °C until use. Phagocytosis assays were performed on 96-well plates containing (per well) 10^7^ CFU of FITC-labeled bacteria and 2 × 10^5^ PMNs (to a multiplicity of infection (MOI) of 200 bacteria per PMN) in RPMI medium or RPMI medium supplemented with 20% human serum.

### 4.10. Microscopy Analysis of Bacterial Phagocytosis in Human Blood

Bacteria from cultures in BHI at log phase (A_550 nm_ 0.3) were harvested via centrifugation (10,000× *g*; 5 min, 4 °C), washed with PBS, gently suspended in 500 µL of heparinized human peripheral blood collected from one healthy donor as previously described [59], under approval of the Committee of the Piracicaba Dental School, State University of Campinas (CEP/FOP-UNICAMP; CAAE 58699622.4.0000.5418). Samples were then incubated at 37 °C for 5 min [75] and duplicate blood smears with aliquots of these suspensions were stained using May–Grunwald Giemsa and analyzed using light microscopy. Digital images were obtained at 1000× total magnification. Three experiments were performed on blood samples collected on different days.

### 4.11. Bacterial Persistence in Human Blood

The survival of *S. sanguinis* strains in human blood was analyzed in ex vivo assays. Briefly, strains were grown in BHI to A_550 nm_ 0.3, harvested via centrifugation (11,000× *g*, 2 min), washed twice in PBS and resuspended in 1 mL of fresh peripheral blood. Aliquots of these suspensions were collected just after bacterial suspension (time 0 h) and after 3 h of incubation at 37 °C under orbital rotation, serially diluted and plated onto BHI agar for the determination of bacterial counts (log of CFU/mL). Similar assays were performed with the difference that blood suspensions were incubated without rotation to allow the PMNs to sink at the bottom of the tube [67]. Three independent experiments were performed in duplicate.

### 4.12. Data Analyses

Biofilm formation and flow cytometry data were compared between strains using Kruskal–Wallis with Dunns’s post hoc test. Pearson’s correlation analysis was applied to compare the amounts of amyloid detected in culture fluids determined in the wild-type strains with previously published data of strain binding to complement proteins and opsonophagocytosis by neutrophils (PMNs). Bacterial viability in human blood were compared using ANOVA with Tukey’s post hoc test.

## Figures and Tables

**Table 1:** Streptococcal strains included in this study.

Strain	Site of Isolation and/or Relevant Characteristics	Source or Reference
*Streptococcus sanguinis*		
SK36	Dental biofilm	ATCC [6]
**SK49** **^†^**	Dental biofilm	Mogens Kilian [69]
SK72 ^†^	Dental biofilm	Mogens Kilian [69]
SK115 ^†^	Dental biofilm	Mogens Kilian [69]
SK169 ^†^	Dental biofilm	Mogens Kilian [69]
SK330 ^†^	Oral cavity	Mogens Kilian [69]
SK353 ^†^	Oral cavity	Mogens Kilian [69]
SK678 ^†^	Blood	Mogens Kilian [69]
SK1056 ^†^	Blood	Mogens Kilian [69]
SKsrtA	∆*ssa_1219*::Erm^r^	This study
SKsrtA	∆*srtA*::Erm^r^; pDL278: *ssa_1219*; Spec^r^	This study
*Streptococcus mutans*		
UA159	Oral isolate, caries-affected Child; serotype *c*	ATCC
OMZ175 ^††^	Dental plaque; serotype *f*; *cnm* ^†^	J. Abranches [70]

^†^ Provided by Dr. Mogens Kilian, Aarhus University, Denmark. ^††^ Provided by Dr. Jacqueline Abranches, University of Florida, U.S.A.

**Table 2:** Oligonucleotides used in this study.

Primer Name	Sequence (Forward/Reverse)	Product Size (bp)
Mutant construction and complementation		
ermE1-AscI ermE2-XhoI	TTGGCGCGCCTGGCGGAAACGTAAAAGAAG TTCTCGAGGGCTCCTTGGAAGCTGTCAGT	979
P1 srtA (SSA_1219P1) P2 srtA (SSA_1219P2-AscI)	CCTATCCAATTACGGCAAGA TTGGCGCGCCCAAGGCTCCTAAGGATGTTC	1430
P3 srtA (SSA_1219P3-XhoI) P4 srtA (SSA_1219P4)	TTCTCGAGGGAAACCTTGCTGACCTGATA TCGCTGGTCTGATGACTGTT	755
C1 srtA (SSA_1219C1-SacI) C2 srtA (SSA_1219-BamHI)	TTGAGCTCGCCCATAATCTGCTCTTTCTG TTGGATCCAAGGCTATGGTAAGCGAACT	1529
RT-qPCR		
16s-RT-F 16s-RT-R	GGAAACTGTTTAACTTGAGTGCA GGCCTAACACCTAGCACTCA	202
srtA-RT-F	TATCAATGCTCAGTGGAAAGC	132
srtA-RT-R	TTTCATCGTACCAGCACCATA	

## Data Availability

The data that support the findings of this study are openly available in https://doi.org/10.25824/redu/M34NUY (accessed on 16 June 2023).

## Supplementary Materials

The supporting information can be downloaded at: https://www.mdpi.com/article/10.3390/ijms242115686/s1.
