# Supplementary material for: Amyloid Fibrils Produced by Streptococcus sanguinis Contribute to Biofilm Formation and Immune Evasion
Source: Int J Mol Sci. 2023 Oct 28;24(21):15686. doi: 10.3390/ijms242115686 (PMC10647432; doi:10.3390/ijms242115686)
Supplement: Supplementary file 1 [file ijms-24-15686-s001.zip › ijms-2675921-supplementary.pdf]

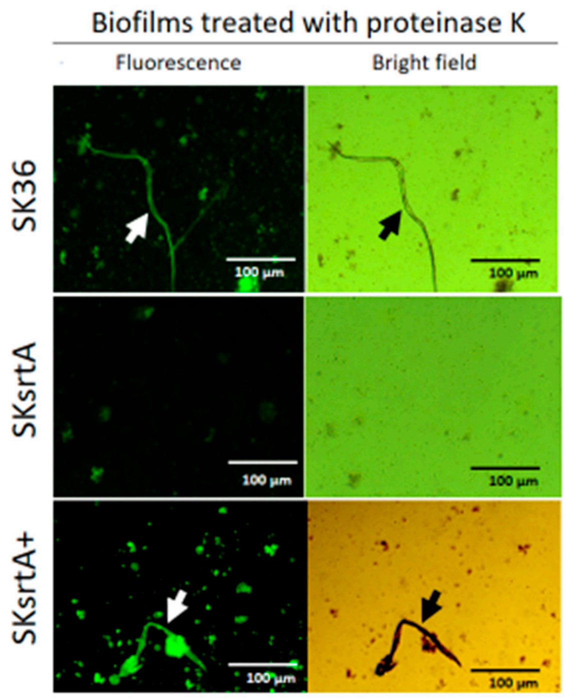

Figure S1. Fluorescence microscopy analysis of *S. sanguinis* biofilms stained with ThT. Biofilms formed over 72 h by strains SK36, SKsrtA and SKsrtA+ in CDM supplemented with 1% sucrose were collected, treated with proteinase K and stained with ThT. Representative images of the biofilms observed by fluorescence (left panels) and light (right panels) microscopy are shown. Arrows indicate amyloid-like aggregates. Digital images were acquired using a fluorescence microscope (Zeiss Axiovrt 40CFL) at 200X total magnification. Strain identities are indicated on the left of the respective panels.
